# Improvement of the observational method for *Plasmodium berghei *oocysts in the midgut of mosquitoes

**DOI:** 10.1186/1756-3305-4-118

**Published:** 2011-06-27

**Authors:** Miho Usui, Shinya Fukumoto, Noboru Inoue, Shin-ichiro Kawazu

**Affiliations:** 1 National Research Center for Protozoan Diseases, Obihiro University of Agriculture and Veterinary Medicine, Inada, Obihiro, Hokkaido 080-8555, Japan

## Abstract

**Background:**

There is a need for improving the method for counting oocysts of *Plasmodium berghei *in the midgut of *Anopheles *mosquitoes. The two methods currently used, the formalin fixation method and the mercurochrome staining method, have contradicting advantages and disadvantages. In the formalin fixation method, the specimen can be preserved but unstained oocysts were often indistinct from the insect tissue. While in the mercurochrome staining method, stained oocysts can be clearly distinguished from insect tissue but the specimen are not well preserved. These two methods were combined in this study to develop a new improved technique in counting the oocysts, in which the specimen can be both stained and preserved well. This technique was evaluated for its accuracy and suitability in observing the oocyst development.

**Findings:**

In the improved technique, the parasite-infected midgut was first stained with mercurochrome, and then fixed with formalin. The specimens were finally observed using light microscopy. To evaluate the accuracy in the oocyst counting with the improved technique, mosquitoes were infected with the green fluorescent protein (GFP)-expressing parasite. Then, the midgut oocysts were counted using both the GFP marker and the improved technique. Results were then compared and showed that the improved technique retrieved 78%-123% (arithmetic mean = 97%) of the oocysts counted using the GFP marker. Furthermore, it was also possible to evaluate the oocyst development with a green filter using the light microscopy.

**Conclusions:**

The improved technique for oocyst counting will be a useful tool for evaluating midgut oocyst numbers and determining the developmental stage of oocysts in parasite-infected mosquitoes.

## Introduction

**Figure 1:**
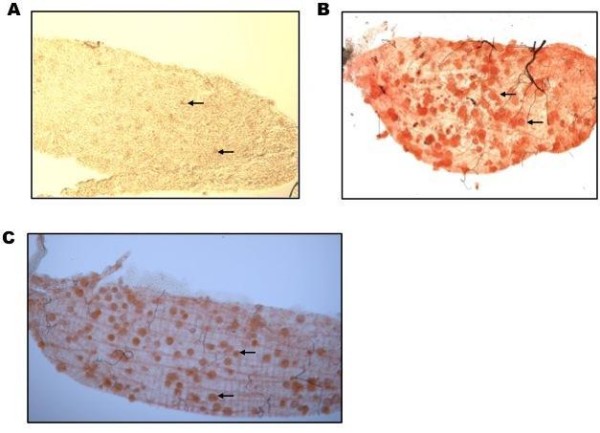
**Comparison of methods currently used for counting oocysts**. (A) In the formalin fixation method, the midgut was opened as a single layered specimen that avoided the overlapping of oocysts. However, unstained oocysts were indistinct and difficult to distinguish from insect tissue. (B) On the other hand, the mercurochrome staining method made the oocysts distinct from the surrounding tissue, although the two layers of oocysts overlapped and they were difficult to distinguish from each other. (C) In the improved technique, the red-stained oocysts could be distinguished clearly from the insect tissue, and in the single layered specimen they were no longer overlapping each other. Arrows indicate oocysts in the midgut of *A. stephensi *at 12-14 days post-feeding of *P. berghei *ANKA strain infected mice.

Malaria is a disease caused by the infection with protozoan parasites of the genus *Plasmodium *and transmitted by *Anopheles *mosquitoes. To study the life cycle of *Plasmodium berghei *in mosquitoes, qualitative and quantitative evaluation of midgut oocysts in experimental infections is needed [1]. There are two methods currently used for counting oocysts. One technique used is the formalin fixation method [2]. In this method, the midgut is fixed with 10% formalin and opened along the median line to prepare a single-layered specimen. The advantage of using this technique is that it can preserve the sample for storage and later observation. However, this technique leaves the oocysts unstained, making them indistinguishable from insect tissues (Figure 1A). Another technique is the mercurochrome staining method [3-5]. In this method, the midgut is stained with 0.5% mercurochrome in water which makes the oocysts be easily distinguished from insect tissues (Figure 1B). However, the resulting specimen from this technique should be observed at the same day since there was no included preservative. This may pose a problem when handling large numbers of mosquitoes for oocyst examination. Furthermore, the observation of bagged (unopened) midguts from a single perspective under the microscope often results in overlapping oocysts leading to inaccuracies in counting. In this study, a new technique for counting oocysts was done by combining the two methods available. This technique produces fixed and stained oocysts in opened midgut specimens which can be utilized for a more reliable counting (Figure 1C).

To evaluate the accuracy of oocyst counting with the improved technique, mosquitoes were infected with the green fluorescent protein (GFP)-expressing parasite and their midguts were dissected for oocyst counting.

## Materials and methods

*P. berghei *ANKA strain which expresses GFP [6] was provided by Mie University, Japan. The GFP expression is derived under HSP70 promoter activity, thus the parasite strain constitutively expresses GFP. The parasite was maintained by mosquito transmission in *A. stephensi *interspersed by a maximum of two serial passages in BALB/c mice (Clea Japan, Tokyo, Japan) [7]. At 12-14 days post-feeding, mosquitoes that had been infected with the parasite strain were dissected, and the midguts were observed under a fluorescence microscope (× 100). The number of oocysts in the midguts was counted using GFP as a marker. After this observation, the midgut samples were stained and fixed with the improved technique for re-counting the oocysts. Briefly, the midgut was stained with 0.5% mercurochrome (Sigma Aldrich Japan Co., Tokyo, Japan) in water at room temperature for 10 min and washed in phosphate buffered saline (PBS) for 10 min (mercurochrome staining method), and then fixed with 10% formalin (Wako Pure Chemical Industries Ltd., Osaka, Japan) for 24 h and opened along the median line (formalin fixation method) for observation. The specimens were observed using light microscopy (× 50 or × 400). The animal experiments in this study were carried out in compliance with the Guide for Animal Experimentation at the Obihiro University of Agriculture and Veterinary Medicine.

## Results and Discussion

The oocysts were counted using both the GFP marker and the improved technique. The oocyst count using the GFP marker ranged between 5 and 272, while with the improved technique, the range was between 5 and 254. In the two independent experiments, counting with the improved technique retrieved 78%-123% (97 ± 2%) of the oocysts that had been counted by GFP (Table 1). Formalin fixation and mercurochrome staining method were also done separately to compare with the GFP marker, yielding 20%-81% (52 ± 3%) and 50%-155% (92 ± 4%) respectively of the oocysts counts as compared with the GFP counting (Table 2). Thirty out of the 32 samples examined using the improved technique retrieved > 85% of the oocysts counted by GFP, and 8 of these samples retrieved > 100%. The counting of > 100% might be attributed to the underestimation of the oocyst number with GFP-based counting because it used an unopened midgut where oocysts were often seen overlapping, and they were sometime difficult to be distinguished even with GFP as marker. The remaining 2 samples only retrieved < 80% of the GFP-counted oocysts and this could have been a consequence of cracking of the oocysts with the cover glass during the GFP-based counting. Another reason for this low counting could have been also due to falsely categorizing agglomerated sporozoites as oocysts during the GFP-based counting. Consequently, the improved technique can produce oocyst count comparable to the GFP-based counting method. We applied the improved technique to the midgut specimens at the earlier phase of the oocyst development. We found that the technique could detect the oocysts at 5 days post-feeding (data not shown). We stored five midgut specimens with 5-160 oocysts stained with the improved technique at room temperature for 2 weeks and recounted the oocyst number. The counts were all similar to those recorded in the initial examinations.

**Table 1:** Number of oocysts counted with GFP and the improved technique.

	No. of mosquitoes	**GFP**^**a**^	**Improved Method**^**b**^	**b/a (%)**^**c**^		No. of mosquitoes	**GFP**^**a**^	**Improved Method**^**b**^	**b/a (%)**^**c**^
	1	272	254	93		1	112	103	92
Exp. 1	2	192	173	90	Exp. 2	2	105	102	97
	3	143	160	112		3	99	94	95
	4	131	118	90		4	70	66	94
	5	80	82	103		5	56	45	80
	6	72	76	106		6	53	51	96
	7	71	70	99		7	53	50	94
	8	71	55	78		8	33	35	106
	9	61	56	92		9	26	32	123
	10	61	52	85		10	22	25	114
	11	57	59	104		11	22	21	96
	12	43	41	95		12	17	17	100
	13	41	45	110		13	14	14	100
	14	8	8	100		14	12	11	92
	15	7	7	100		15	8	8	100
	16	5	5	100		16	6	6	100

^a ^Number of oocyst counted with GFP.

^b ^Number of oocyst counted with the improved method.

^c ^Retrieved oocyst count (a/b) = number of oocyst counted with the improved method/number of oocyst counted with GFP × 100 (%). Arithmetic mean ± S.E.M. of the retrieved oocyst count from 32 mosquitoes was 97 ± 2 (%).

**Table 2:** Number of oocysts counted with GFP and the methods currently used for counting oocysts.

Method	Number of dissected mosquitoes	Retrieved oocyst count (%)^a^
Formalin-method	36	52 ± 3 (20-81)
Mercurochrome-method	36	94 ± 4 (50-155)

^a ^Retrieved oocyst count = number of oocyst counted with formalin fixation method or mercurochrome staining method/number of oocyst counted with GFP × 100 (%). Arithmetic mean ± S.E.M. (range of retrieved oocyst counts) from 36 mosquitoes were shown.

In addition, the specimen (red-stained oocysts) observation in improved technique under light microscopy with green filter (GFP-ET, BP 470/40 nm, Lica Japan, Tokyo, Japan) allows differentiation between immature oocysts contained sporoblasts and mature oocysts filled with needle-shaped sporozoites (data not shown).

## Conclusion

Accurate evaluation of the infection rate of midgut oocysts is important for understanding the relationship between vectors and parasites in experimental infections. The improved technique for oocyst counting will be a useful tool for evaluating midgut oocyst count and determining the developmental stage of oocysts in parasite-infected mosquitoes.

## Competing interests

The authors declare that they have no competing interests.

## Authors' contributions

MU: designed and performed the experiments. Redacted the manuscript. SF, NI and SIK: contributed to the study design. Edited the manuscript. All authors read and approved the final manuscript.
